# Observation and control of the weak topological insulator state in ZrTe_5_

**DOI:** 10.1038/s41467-020-20564-8

**Published:** 2021-01-18

**Authors:** Peng Zhang, Ryo Noguchi, Kenta Kuroda, Chun Lin, Kaishu Kawaguchi, Koichiro Yaji, Ayumi Harasawa, Mikk Lippmaa, Simin Nie, Hongming Weng, V. Kandyba, A. Giampietri, A. Barinov, Qiang Li, G. D. Gu, Shik Shin, Takeshi Kondo

**Affiliations:** 1 Institute for Solid State Physics, University of Tokyo, Kashiwa, Chiba 277-8581 Japan; 2 Research Center for Advanced Measurement and Characterization, National Institute for Materials Science, Tsukuba, Ibaraki 305-0003 Japan; 3 Department of Materials Science and Engineering, Stanford University, Stanford, CA 94305 USA; 4 Beijing National Laboratory for Condensed Matter Physics and Institute of Physics, Chinese Academy of Sciences, Beijing, 100190 China; 5 Elettra - Sincrotrone Trieste, Basovizza, Italy; 6 Department of Physics and Astronomy, Stony Brook University, Stony Brook, NY 11794 USA; 7 Condensed Matter Physics and Materials Science Department, Brookhaven National Laboratory, Upton, NY 11973 USA; 8 Office of University Professor, University of Tokyo, Kashiwa, Chiba 277-8581 Japan; 9 Trans-scale Quantum Science Institute, University of Tokyo, Bunkyo-ku, Tokyo 113-0033 Japan

**Keywords:** Electronic properties and materials, Surfaces, interfaces and thin films, Topological insulators

## Abstract

A quantum spin Hall (QSH) insulator hosts topological states at the one-dimensional (1D) edge, along which backscattering by nonmagnetic impurities is strictly prohibited. Its 3D analogue, a weak topological insulator (WTI), possesses similar quasi-1D topological states confined at side surfaces. The enhanced confinement could provide a route for dissipationless current and better advantages for applications relative to strong topological insulators (STIs). However, the topological side surface is usually not cleavable and is thus hard to observe. Here, we visualize the topological states of the WTI candidate ZrTe_5_ by spin and angle-resolved photoemission spectroscopy (ARPES): a quasi-1D band with spin-momentum locking was revealed on the side surface. We further demonstrate that the bulk band gap is controlled by external strain, realizing a more stable WTI state or an ideal Dirac semimetal (DS) state. The highly directional spin-current and the tunable band gap in ZrTe_5_ will provide an excellent platform for applications.

## Abstract (web summary)

Topological side surface, characterization of a weak topological insulator (WTI), has rarely been investigated. Here, Zhang et al. visualize a quasi-one dimensional, spin-momentum locked band on the side surface of the WTI candidate ZrTe_5_, and manipulate the bulk band gap by strain.

## Introduction

Two of the most prominent examples of topological insulators in three dimensions are the STIs and WTIs^1–4^. Among them, STIs have been widely studied in the past decades both in theories and experiments. They host 2D spin-momentum locked Dirac cones on all surfaces, in which the perfect backscattering is prohibited, while general scattering off 180° still exists^5,6^. On the other hand, WTIs host surface states only on particular side surfaces. They were thought to be weak (or not robust) since two adjacent layers in even-layer WTIs may couple with each other, leading to a topologically trivial phase. However, it was later found that the surface states of WTIs are actually robust owing to the delocalization of surface electrons^7^. The weak interlayer coupling in WTIs generally yields a topological surface state with quasi-1D dispersion; this could prohibit even general scattering off 180° to establish dissipationless spin current, which cannot be realized in STIs. Even with such advantages, theoretical proposals of WTIs are rare^8–15^, and experimental investigations of the topological side surfaces are very challenging; layered WTI materials are inherently cleavable only on the top surface, and thus there is difficulty in preparing a large and uniform side surface for observation. One exception is Bi_4_I_4_, where top and side planes are both naturally cleavable^14^. However, such crystal structures may pose obstacles in preparing a single side surface with the topological feature for applications.

The material which has been best studied from the early stage of exploring a WTI among scarce candidates is ZrTe_5_, which consists of a van der Waals layered structure; notably, this compound exhibits very high mobility^16^, thus it has been regarded as a promising platform for devices. Nonetheless, the bulk topology of ZrTe_5_ has not been experimentally identified to date because the observation of band structure on the side surface has not been successful so far. All the previous surface-sensitive studies were carried out on the top surface^17–20^, which only confirms the lack of surface states; whether or not this material is topological, therefore, has not been determined beyond speculation. Another difficulty for this study is that ZrTe_5_ is in proximity to multiple topological phases, whereas this feature could bring an attractive functionality of controlling bulk topology by fine-tuning a physical parameter^10^.

## Results

**Fig. 1:**
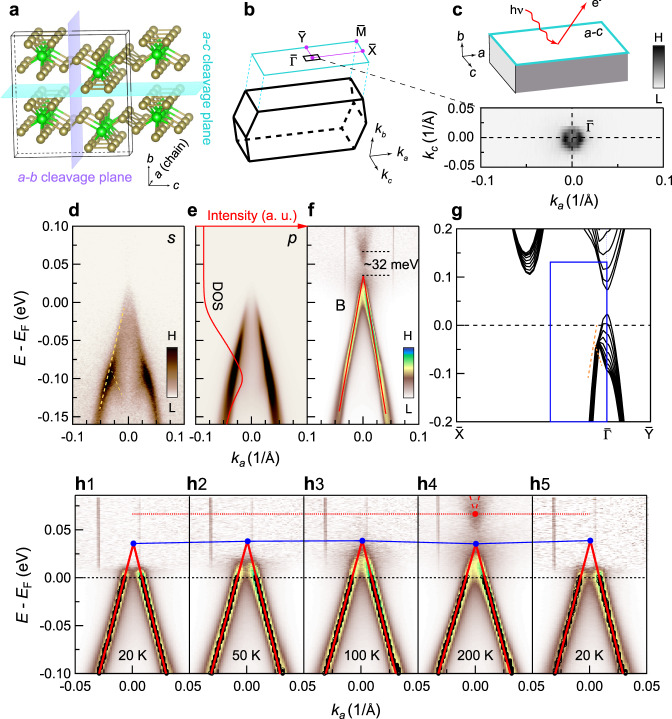
Band structure of the top *a-c* surface. **a** Crystal structure of ZrTe_5_ and its cleavage planes. **b** The bulk Brillouin zone (BZ) and its projected surface BZ on the top *a-c* surface. **c** Fermi surface measured from the *a-c* surface, of which the range is indicated by the black box in **b**. **d**–**e** Band structure measured from the *a-c* surface with *s* and *p*-polarized light at 200 K, respectively. Light-yellow dashed lines in **d** highlight the band splitting around −0.1 eV. The red line in **e** shows the density of states (DOS). **f** Same as **e**, but normalized with its DOS for a clear presentation of the conduction band above Fermi level (*E*_F_), which will not affect the MDC peak positions. Details of the normalization can be found in Supplementary Fig. 2. **g** Band structure from first-principles slab calculations on the *a-c* surface. The blue box roughly indicates the momentum range in **d**–**f**. Orange dashed lines highlight the splitting similar to the one in **d**. **h** Temperature dependence of the band structure taken with *p*-polarized light. The black markers are extracted from MDC peaks, and the red solid lines are the fitting results of the black markers. The blue line with solid markers indicates the top of the valence band at different temperatures. The red dashed line marks the conductance band bottom at 200 K. Details of the band fitting can be found in “Methods” section.

ZrTe_5_ has a quasi-1D crystal structure. The lattice constant *a* along the chain direction is ~4.0 Å. Except for the chain direction, the lattice constants for the other two directions are both large, as shown in Fig. 1a: The layer distance along the *b* direction is ~7.3 Å, and it is ~6.9 Å along the *c* direction^21^. As a result of the large layer distances, in principle, it should be possible to cleave both surfaces. Along the *b* direction, the layers are stacked by van der Waals forces, and a clean surface can be easily obtained by cleavage. Instead, Te–Te bonding exists between adjacent layers along the *c* direction, causing much more difficulty in cleaving the *a-b* surface. All the surface-sensitive studies reported so far have been carried out on the *a-c* surface, which is easy to cleave^17–20,22,23^.

### Band structure of the top surface

We observed a tiny hole-type Fermi surface on the *a-c* surface, as shown in Fig. 1c. The band along *k*_*a*_ (Fig. 1d–e) shows a cone-shape dispersion, similar to that in the previous reports except for the difference on Fermi level (*E*_F_)^17,19,22^. We notice a splitting of the band in Fig. 1d at approximately −0.1 eV, with one branch going up and the other going down, as indicated by the dashed yellows lines in Fig. 1d. Such a feature most likely originates from a *k*_*z*_ average effect, and it is indeed reproduced by the first-principles slab calculations plotted in Fig. 1g along $\overline{{{\Gamma }}}\overline{X}$, where the series of bands come from different *k*_*z*_ (*k*_*b*_); this further indicates that the slab calculations are reliable to explain the ARPES data. The measurements with *p*-polarized light (Fig. 1e) reveal only the cone-shaped branch going up, which is thus suitable for the study of the bulk band gap.

To have a better visualization of the band structure over the whole energy range, the data in Fig. 1e is normalized by its density of states (integration of EDCs over *k*_*a*_), as plotted in Fig. 1f (More details can be found in Supplementary Fig. 2). A small band gap with the size of about 32 meV is observed in the data at 200 K. Our experiments over a temperature cycle of 20 K-200 K-20 K (Fig. 1h) have confirmed almost no change in the valence band at different temperatures; this result clearly differs from the previous reports demonstrating a large energy shift in the band with temperature^17,19,20,24^. On the other hand, we have confirmed a band shift in an ARPES system with a worse vacuum level (~7 × 10^−11^ Torr *v*. *s*. ~1 × 10^−11^ Torr for Fig. 1h); the band shift actually occurs even for the sample kept at the same temperature (See Supplementary Fig. 1 for more details). These results lead us to conclude that ZrTe_5_ should be in a stable phase, which is not a strong topological insulator since surface states are absent in the data, but either a normal insulator or a WTI. However, with the data merely from the top surface, it is impossible to distinguish the two phases experimentally.

### Band structure of the side surface

**Fig. 2:**
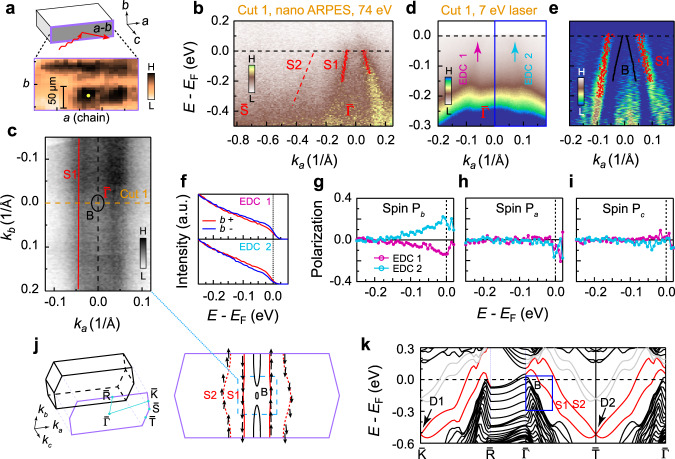
Surface band of the side *a-b* surface. **a** Real-space photoemission intensity mapping from a synchrotron-based nano-ARPES. The width of the cleaved area with high intensities of photoelectrons is about 50 μm. **b** Band structure from nano-ARPES with 74 eV photons at the position indicated by yellow solid dot in **a**. The red dots are duplicates of the ones in **e**, and the red-dashed line roughly indicates the band S2 in **k**. **c** Fermi surface measured from the *a-b* surface with laser-based ARPES. **d** Band structure of Cut 1 in **c**. The blue box corresponds to the one in **k**. **e** MDC curvature^38^ plot of **d**. The red dots are extracted from the MDC peaks in **d**, and the black dots (band B) are duplicates of the black markers in Fig. 1h4. **f** Spin-resolved EDCs (along *b* direction) at EDC 1 and EDC 2 indicated in **d**, respectively. **g**–**i** Spin polarization curves along *a*, *b* and *c* directions at EDC 1 and EDC 2, respectively. More details on the spin data analysis can be found in Supplementary Fig. 4. **j** Projected surface BZ and Fermi surface from first-principles slab calculations on the *a-b* surface. The black arrows indicate the in-plane spin direction. **k** Band structure from first-principles slab calculations on the *a-b* surface. The red lines indicate the surface bands, while the black lines represent the bulk bands. Due to the presence of the broken bonds at the surface, two trivial bands colored in gray appear below *E*_F_ in first-principles slab calculations. They are not intrinsic and do not appear in the calculations based on Wannier functions^10,26^, of which the Fermi surfaces are not shown in **j**. The blue box corresponds to the one in **d**.

To identify the topological phase of ZrTe_5_, the observation of the side *a-b* surface is essential. We successfully cleaved the side surface with a top post and silver epoxy. The problem, however, is that the cleaved areas are very small and inhomogeneous. With a synchrotron-based nano-ARPES (spot size < 1 μm), we took real space maps of photoemission intensity on several samples, and found that the typical dimension of cleaved areas is around or smaller than 50 μm (Fig. 2a). Several positions of one cleaved area are measured and they all give the same band structure, as shown in Supplementary Fig. 3, indicating that the cleaved area with high photoelectron intensities is homogeneous. The selective measurements only of such a small surface area are beyond the capacity of most ARPES systems. Nevertheless, our laser-based micro-ARPES (spot size 50 μm) resolved this difficulty and clearly revealed the band structure on the side *a-b* surface. A quasi-1D Fermi surface (S1) is exhibited in Fig. 2c by plotting ARPES intensities about the Fermi level. The band S1 shows a hole-like dispersion along *k*_*a*_ (Fig. 2d–e), which has a Fermi vector *k*_*F*_ much larger than that of the band B observed near $\overline{{{\Gamma }}}$ on the *a-c* surface (Fig. 1f). The band S1 is, thus, distinct from the bulk band B located at the center of the BZ ($\overline{{{\Gamma }}}$). The bulk band B cannot be identified in ARPES data on the side *a-b* surface likely due to a much weaker intensity than that of the surface states. The synchrotron-based nano-ARPES measurements for Cut1 with a different photon energy (Fig. 2b) show similar result to the data obtained by laser-based ARPES: Only the band S1 was clearly observed, and its dispersion is almost identical to that of the laser-based ARPES data. This coincidence supports that the band S1 has a surface origin.

The spin polarization is expected for the topological surface state. To confirm this, we have used spin-resolved ARPES. In Fig. 2f, we plot EDCs (EDC 1 and EDC 2; see Fig. 2d) which are spin-resolved in the *b*-direction; a clear spin polarization is detected (Fig. 2g). In contrast, the spin polarization is almost zero along the *a* and *c* directions (Fig. 2h–i). Therefore, the band S1 is spin-polarized along the *b*-direction with spin-momentum locking, forming a typical spin-texture of WTIs with quasi-1D dispersion (see Fig. 2j).

We also carried out the first-principles slab calculations on the side *a-b* surface to fully understand the ARPES data. The calculated Fermi surface (Fig. 2j) displays four sheets: two open sheets (red color) and two closed sheets (black color). By comparing these calculations with our ARPES data (Fig. 2c), we ascribe the inner open sheet to the band S1, and the closed sheets to the bulk band B. The outer open sheet (S2) was not observed with either laser-based ARPES or synchrotron-based ARPES most likely due to a small photoemission cross section. In the slab calculations, the bands S1 and S2 are localized at the surface and have spin polarization indicated by the black arrows in Fig. 2j; the spin orientation of the band S1 is consistent with our data of spin-resolved ARPES (Fig. 2f–i). The slab calculations (Fig. 2k) clearly illustrate the topological nature of the bands S1 and S2, which connect the valence and conduction bands and cross with each other at two BZ boundary points, $\overline{K}$ and $\overline{T}$; The formation of two Dirac points is a typical feature of WTIs^1,2^. Although the Dirac points have not been directly detected, our data demonstrating the quasi-1D spin-momentum locking and its agreement with the first-principles calculations strongly support that the band S1 originates from the topological surface state of a WTI. The trivial bands (colored gray in Fig. 2k) crossing *E*_F_ in the calculations are from the dangling bonds at the *a-b* surface, which will be removed in the calculations by proper atom absorption^25^ and do not appear in the surface Greens’ function calculations^10,26^ insensitive to the dangling bonds. They are not intrinsic, and not observed in the experiments.

### Band structure with external strain

The small bulk gap (~32 meV) we observed by ARPES indicates that the WTI state in ZrTe_5_ is protected only marginally from external perturbations. This is compatible with transport measurements showing semimetal behaviors in ZrTe_5_^22,27–30^. Theoretical calculations proposed that the band gap changes with external strain^10^. Such effect could be adopted to stabilize the topological state of matter in WTI, DS, or even STI state; a relevant feature has been previously reported with the data showing a strain-dependent magneto-transport possibly caused by a band gap variation^31^. However, the signature of transport measurements is indirect, calling for the direct evidence of strain effect on the band structure by ARPES measurements^32^.

**Fig. 3:**
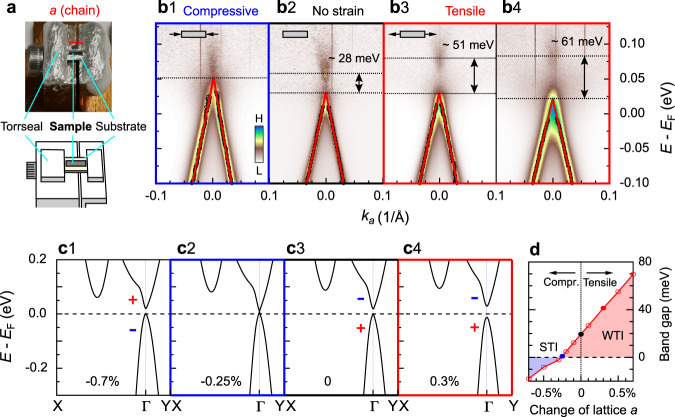
Control of the topological phase by external strain. **a** The strain device. A screw is used to compress or stretch the substrate (and thus the sample attached to it) along the chain direction of the samples. The red line indicates the chain direction. **b** Bulk band gap change with compressive and tensile strain. With compressive strain (b1), the gap is (nearly) closed, reaching a Dirac semimetal state. With tensile strain (b3–b4), the band gap becomes larger, stabilizing the WTI state. The data are taken with *p*-polarized photons and normalized by their density of states (DOS). The black markers are extracted from the MDC peaks, and the red solid lines are the fitting results of the black markers, same as the ones in Fig. 1h. **c** Calculations on the band structure with different lattice constant *a*. + and − signs indicate the parity of the two bands. **b**, **c** The blue (red) frames correspond to compressive (tensile) strain. **d** Calculated phase diagram with different lattice constant (strain). Blue, black, and red solid markers roughly indicate the experimental values in **b**.

In this study, the samples are glued on Ti or BeCu substrates, to which compressive or tensile strain is applied along the chain direction of ZrTe_5_ (Fig. 3a; see “Methods” section for more details). The band structures under different strain are displayed in Fig. 3b. The sample with no strain shows a band gap of ~28 meV (Fig. 3b2), similar to that in Fig. 1. We have applied tensile strain to two samples, and find an increase of the band gap up to more than the double of the original value (~61 meV; Fig. 3b4), which makes a WTI state much more robust to external perturbations. In contrast, we could instead reduce the gap value by the compressive strain, even realizing a Dirac semimetal state with a gap no longer distinguishable (Fig. 3b1). We found that the cleaving process causes wrinkles in the sample under large compressive strain to relax the stress on the crystal lattice. It thus becomes harder to apply larger compressive strain to realize a STI state by ARPES, which requires the sample cleavage. We note, however, that cleavage is not necessary for most applications, thus a STI state could be easily reached by a compression method. In Supplementary Fig. 6, we further show that the band gap can be reversibly controlled by external strain.

To better understand our data, we have carried out first-principles calculations of the band structure for different lattice constants of *a* (Fig. 3c); In ARPES, the strain was applied to the samples along the chain direction (the *a*-axis). The calculations for compression by 0.25% (Fig. 3c2) obtain a DS state, which is consistent with the ARPES results under compressive strain in Fig. 3b1. On the other hand, the case of lattice expansion by 0.3% (Fig. 3c4) corresponds to our data under tensile strain in Fig. 3b3. Hence, we conclude that about 0.3% compression and stretch are reached in our experiments with a strain device. This is consistent with the strain values measured by X-ray diffraction on the sample and strain gauge on the substrate (See Supplementary Fig. 5). The band gap variation with the lattice constant *a* is summarized in Fig. 3d, where the experimental data roughly correspond to the solid dots. The calculations further indicate that a STI state with a gap of 18 meV can be reached with 0.7% compression (Fig. 3c1), and a WTI state with a band gap about 70 meV can be reached with 0.7% stretch. Our experimental results provide the direct evidence with ARPES on the band gap control and topological phase transition by external strain.

## Discussion

**Fig. 4:**
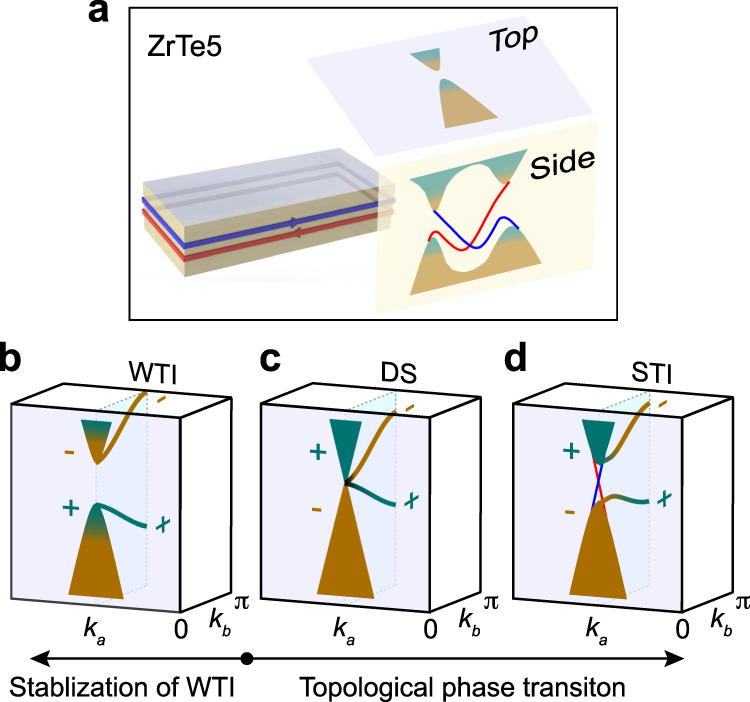
Weak topological insulator state of ZrTe_5_ and control of its topological phase. **a** In ZrTe_5_, there are surface states on the side surface, while no surface state exists on the top surface. **b**–**d** Topological phase transition in ZrTe_5_. The WTI state of ZrTe_5_ can be stabilized by tensile strain (**b**), and a topological phase transition to DS (**c**) or even STI (**d**) can be obtained by compressive strain. There are two band inversions at k_*b*_ = 0 and k_*b*_ = *π* in the WTI state of ZrTe_5_. The band inversion at k_*b*_ = 0 is switched by external strain in **b**–**d**, while the band inversion at k_*b*_ = *π* has no change.

In summary, we have revealed that ZrTe_5_ is a WTI, as sketched in Fig. 4a, by directly observing the electronic structure not only on the top surface but also on the side surface of crystal. On the top *a-c* surface of ZrTe_5_, the band structure was confirmed to be gapped at any temperatures lower than 200 K. In contrast, the side *a-b* surface exhibits quasi-1D surface band with spin-momentum locked texture, which illustrates the WTI nature of ZrTe_5_ distinct from a STI or a normal insulator. Under external strain, the band gap increases with tensile strain and decreases with compressive strain, providing a way to control the bulk gap of the WTI state, or to realize a topological phase transition to a Dirac semimetal state, or even a STI state (Fig. 4b–d). A large band gap obtained by tensile strain can bring further protection of the 3D WTI state or monolayer QSH states in ZrTe_5_ from thermal fluctuations or other external perturbations^33^. The quasi-1D surface states yielding the highly directional dissipationless spin current and the strain-tunable bulk gap make ZrTe_5_ an ideal platform for 2D devices and spin engineering.

## Methods

### Sample growth

High-quality crystals of ZrTe_5_ were synthesized by the flux method with Te as the flux. High-purity elements (99.99999% Te and 99.9999% Zr) were loaded into a double-walled quartz ampoule and sealed under vacuum. The materials were first melted at 900 °C in a box furnace and fully rocked for 72 h to achieve a homogeneous mixture. The melt was then slowly cooled and rapidly heated between 445 and 505 °C for 21 days. Needle-like crystals were obtained.

### ARPES

Laser-based ARPES and spin-resolved ARPES measurements were performed at the Institute for Solid State Physics, the University of Tokyo, with a laser delivering 6.994-eV photons. Photoelectrons were detected with ScientaOmicron R4000 analyzer and DA30L analyzer. The angle resolution was 0.3° (0.7°) and the overall energy resolution was set to ~5 meV (30 meV) in ARPES (spin-resolved ARPES) measurements. Three-dimensional spin-polarizations of photoelectrons were detected by two VLEED-type spin detectors^34^. Synchrotron-based nano-ARPES measurements were performed at the 3.2L-Spectromicroscopy beamline of the Elettra Light Source. A Schwarzschild objective was used to focus the photon beam to a spot <1 μm in size. The photon energy was set to 74 eV and temperature was set to around 100 K. The overall energy resolution was set to be better than 60 meV.

### Band fitting

The band dispersions are extracted from MDC peaks. To avoid any complication, we simply used the position of the MDC maximums. Since the band dispersion highly resembles a cone structure, we used the following formula to fit the band structure:

1 $$f(x)\,=\,\left\{\begin{array}{ll}{c}_{1}(x\,-\,c_4)\,+\,{c}_{3}, & x\,\ge\, {c}_{4}\\ {c}_{2} (x\,-\,c_4)\,+\,{c}_{3}, & x\,<\,{c}_{4}\end{array}\right.$$

where *c*_1_, *c*_2_, *c*_3_, *c*_4_ are the fitting parameters. If the computer program returns 0 or 1 for the inequality *x* ≥ *c*_4_ and *x* < *c*_4_, the above formula can be rewritten in one line:

2 $$f(x)\,=\, [{c}_{1}(x\,-\,c_4)\,+\, {c}_{3}] (x\,\ge\, {c}_{4})\,+\, [{c}_{2}(x\,-\,c_4)\,+\,{c}_{3}](x{\,}< {\,}{c}_{4})$$

Since the band dispersion may not be perfectly symmetric, two independent slopes were used for a better fitting. We note this method will underestimate the gap size, as the band top is actually curved due to the band gap opening. However, since the band gap is very small and the band dispersion resembles a cone structure to the very top, the difference should be quite small. Also, we mainly compare the gap change with strain, such underestimation should not affect the comparison.

### Strain device

The strain device shown in Fig. 3a has a unibody structure made of BeCu. There are two walls on the flat plate: a thick one (left) and a thin one (right). A substrate is fixed on the top of the two walls. By reducing or increasing the space between the two walls, we can apply compressive or tensile strain to the substrate, thus to the sample. In the device to apply compressive strain, the thick wall (left) has a through hole, while the thin wall (right) has a threaded hole. Thus by tightening the screw, the space between the two walls will be reduced, and compressive strain will be applied. In the device to apply tensile strain, the thick wall has a threaded hole, while the thin wall is blinded. Thus by the tightening the screw, the space will be enlarged, and tensile strain will be applied. Due to the limited space of the sample holder, Torr Seal is used to fix the substrate on top of the walls.

The data in Fig. 3b are all taken from one thick sample. We exfoliated this thick sample with scotch tape and got different flakes. Then we mounted these different flakes to the substrates of the strain devices, and carried out the experiments. With this method, we avoided the problem that different samples may have slightly different bulk band gap.

### DFT calculations

Our calculations have been performed with the projector augmented wave method implemented in Vienna ab initio simulation package^35,36^. Generalized gradient approximation of Perdew-Burke-Ernzerhof type is used^37^. The k-point sampling grids are set to 13 × 13 × 7 and 7 × 7 × 1 for ZrTe_5_ bulk and ZrTe_5_ slab, respectively. Spin-orbit coupling is included in the calculations. Considering the fact that ZrTe_5_ is a strong topological insulator^10^ when the experimental lattice constants^21^ are used, we take a slightly increased interlayer distance (0.25 Å) to mimic the weak topological insulator phase, which is consistent with our experimental observations. The ionic positions are relaxed until force on each ion is less than 0.01 eV/Å.

## Supplementary information

Supplementary Information

## Data Availability

The data that support the findings of this study are available from the corresponding authors upon reasonable request.
