# Supplementary material for: Observation and control of the weak topological insulator state in ZrTe5
Source: Nat Commun. 2021 Jan 18;12:406. doi: 10.1038/s41467-020-20564-8 (PMC7813838; doi:10.1038/s41467-020-20564-8)
Supplement: Supplementary file 1 — Supplementary Information [file 41467_2020_20564_MOESM1_ESM.pdf]

Supplementary Information for "Observation and control of the weak topological insulator state in $ZrTe_5$"

Peng Zhang,[1,*] Ryo Noguchi,[1] Kenta Kuroda,[1] Chun Lin,[1] Kaishu Kawaguchi,[1] Koichiro Yaji,[2] Ayumi Harasawa,[1] Mikk Lippmaa,[1] Simin Nie,[3] Hongming Weng,[4] V. Kandyba,[5] A. Giampietri,[5] A. Barinov,[5] Qiang Li,[6] G.D. Gu,[6] Shik Shin,[7,1] and Takeshi Kondo[1,†]

[1]*Institute for Solid State Physics, University of Tokyo, Kashiwa, Chiba 277-8581, Japan*
[2]*Research Center for Advanced Measurement and Characterization, National Institute for Materials Science, Tsukuba, Ibaraki 305-0003, Japan*
[3]*Department of Materials Science and Engineering, Stanford University, Stanford, California 94305, USA*
[4]*Beijing National Laboratory for Condensed Matter Physics and Institute of Physics, Chinese Academy of Sciences, Beijing 100190, China*
[5]*Elettra - Sincrotrone Trieste, Basovizza, Italy*
[6]*Condensed Matter Physics and Materials Science Department, Brookhaven National Laboratory, Upton, NY, USA*
[7]*Office of University Professor, University of Tokyo, Kashiwa, Chiba 277-8581, Japan*

* zhangpeng@issp.u-tokyo.ac.jp
† kondo1215@issp.u-tokyo.ac.jp

Supplementary Note 1: Band shift with time in low vacuum

The data in Fig. 1 was taken from an ARPES system with a vacuum better than 1×10^{-11} Torr. No band shift at the same temperature was observed. However, we also carried out experiments at an ARPES system with lower vacuum ($\sim 7 \times 10^{-11}$ Torr). With this system, we noticed a band shift with time, even at the same temperature. In Supplementary Fig. 1a - 1d, we displayed a data sequence taken at different time but under the same experimental conditions. The band shift is summarized in Supplementary Fig. 1e. It is obvious that the band shifts toward lower binding energy, showing a hole doping effect. We observed the same phenomenon for different samples.

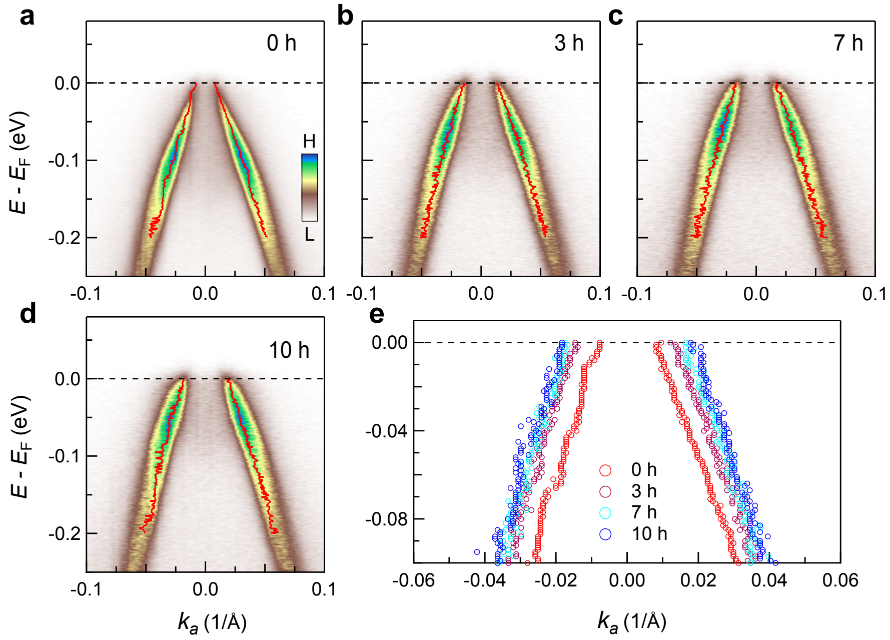

Supplementary Figure 1. **Time-dependent band shift.** (a - d) Band structure taken under the same conditions but at different time. Temperature is set to 50K, and a 7-eV laser is used. Red lines are extracted from the MDC peaks. (e) Comparison of the band dispersions extracted in (a - d). The band shows a hole doping effect as the experiment time increases.

Supplementary Note 2: Data normalization with density of states

The raw data divided by Fermi function at 200K is shown in Supplementary Fig. 2b - 2c. The density of states (DOS, integration of EDCs over k_a) of Supplementary Fig. 2c roughly shows a linear decrease toward the conical point (Supplementary Fig. 2d), which is very different from the behavior of an ideal linear dispersion. For a linear dispersion at specific k_{b0} and k_{c0}, $E(k_a, k_{b0}, k_{c0}) \sim ak_a + b$, in calculation the integration over k_a gives a constant ($\rho(E) = dN/dE \sim dk_a/dE \sim constant$), as shown in Supplementary Fig. 2g - 2h. The decreased intensity in experimental data should come from the matrix element effect. As the intensity near the conical point is rather weak, the decreased intensity makes it very difficult to obtain the conical points of the valence and conduction bands. To remove such intensity change induced by matrix element effect, the raw data is normalized by its DOS. The resulting constant intensity will be similar to that from the calculation. The result is shown in Supplementary Fig. 2e. The gap size obtained from this data should be more precise.

Furthermore, the feature of the EDC at $k_a = 0$ from Supplementary Fig. 2e is more consistent with the calculations than that from Supplementary Fig. 2c. As shown in Supplementary Fig. 2h, the peak of the EDC at $k_a = 0$ is pronounced and indicates the position the conical point. In Supplementary Fig. 2f we compared the EDCs at $k_a = 0$ from Supplementary Fig. 2c and 2e. The EDC from the data divided by Fermi function shows no peak feature, different from the calculation in Supplementary Fig. 2h. Instead, the EDC from the data normalized by DOS has a well-defined peak, similar to the calculations. Thus, we use this peak as the band bottom of the conduction band. The gap size obtained by this method is comparable with the data reported in Ref. [1, 2].

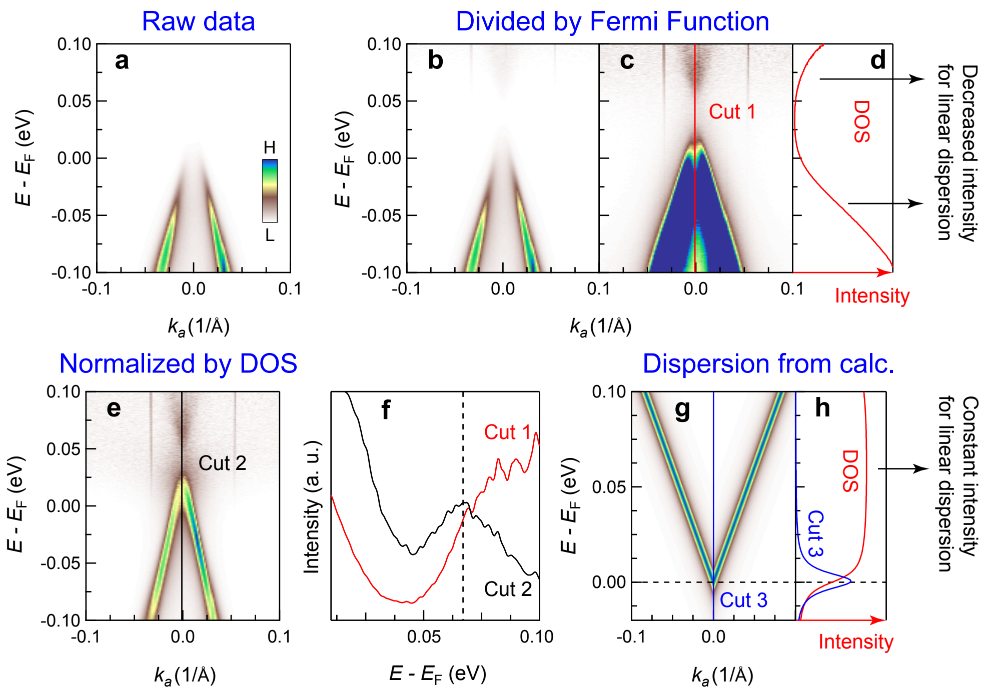

Supplementary Figure 2. **Data normalization with DOS.** (a) Intensity plot of the raw data at 200K, same as Fig. 1(e). (b) Intensity plot of the raw data divided by Fermi function. (c) Same as (b), but the intensity was scaled up to show the conduction band. (d) The DOS of the data in (c). The DOS is obtained by integrating EDCs over k_a. (e) Intensity plot of the raw data normalized by its DOS. (f) EDCs at $k_a = 0$ in (c) and (e). (g) Intensity plot of a linear dispersion from simulation. (h) The EDC at $k_a = 0$ and DOS of (g).

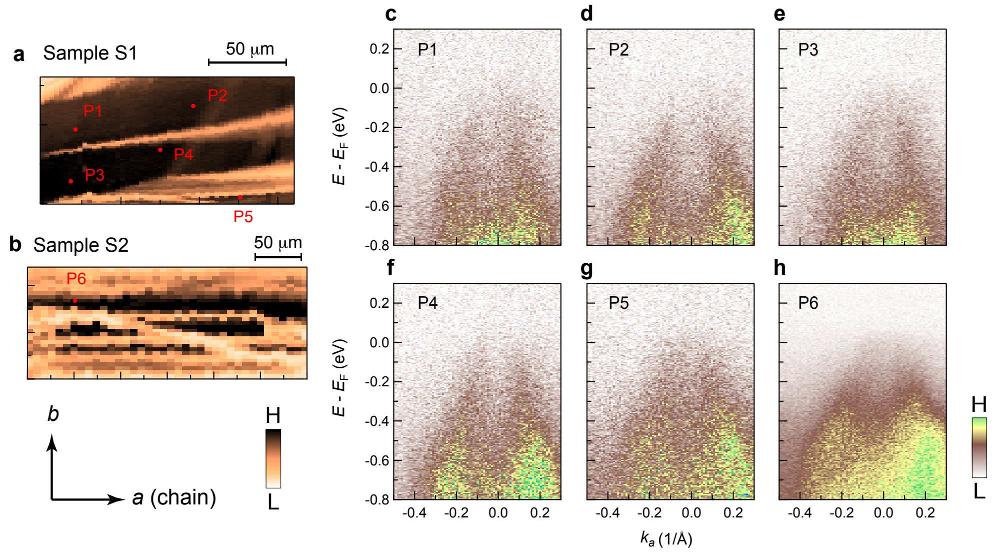

Supplementary Figure 3. **Homogeneity of the cleaved *a*-*b* surface.** (a - b) Real space intensity mapping of the *a*-*b* surfaces from two different samples. (c - h) Band structure measured from different positions indicated by red solid dots in (a) and (b).

Supplementary Note 3: Homogeneity of the cleaved side surface

Our measurements with the synchrotron-based nano-ARPES revealed that the successfully cleaved areas with high photoelectron intensities are very small (~50 μm in width) in the $a - b$ plane. Here we demonstrate that the electronic states within such small cleavage areas are rather homogenous. This fact is particularly important to justify the reliability of our data taken by laser-ARPES with a relatively large photon beam ($\sim$ 50 μm).

Supplementary Figure 3 exhibits the band dispersions measured by nano-ARPES at many different points on the cleaved areas with high intensities from two pieces of the sample. For the sample S1, the ARPES measurements were carried out on five different points marked by the red solid dots in Supplementary Fig. 3a , and the corresponding band structures are shown in Supplementary Fig. 3c - 3g. We also got signal from another piece of the sample (sample S2), and display its band structure in Supplementary Fig. 3h. Significantly, all these measurements give basically the same band structure, indicating that the electronic states are homogenous within the successfully cleaved areas. This justifies that the intrinsic electronic structure for the side surface of $ZrTe_5$ can be also obtained by laser-ARPES with laser spot of 50 μm in size, which is similar to the typical width of the successfully cleaved areas with homogenous electronic states.

Supplementary Note 4: Spin polarization calibration

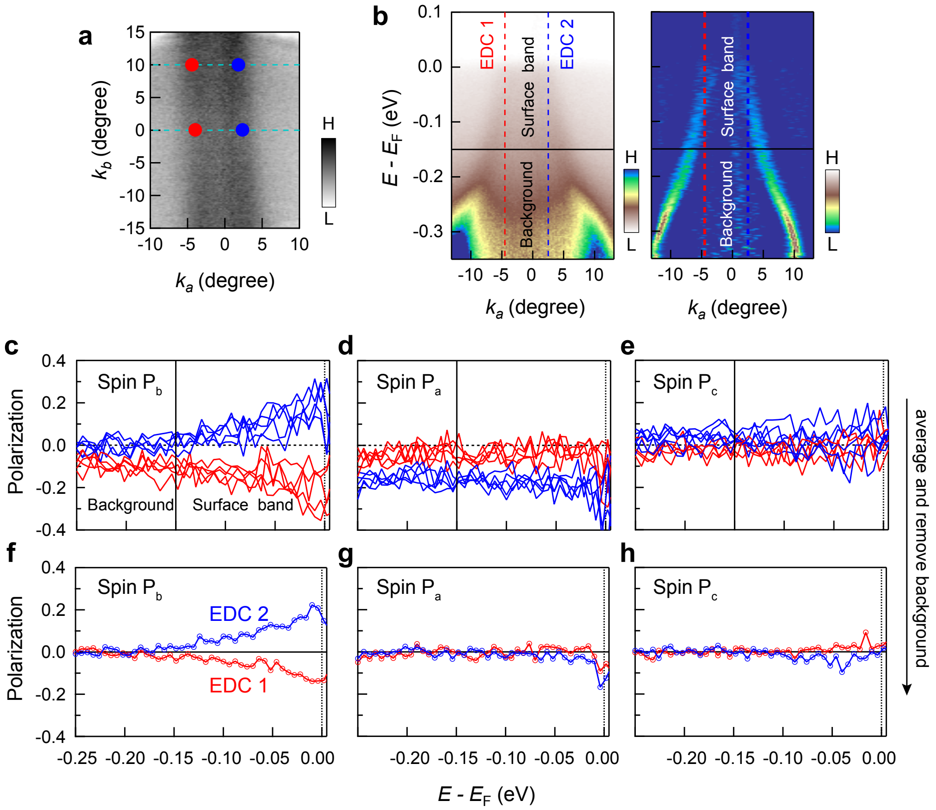

Supplementary Figure 4. **Spin polarization calibration.** (a) Fermi surface of the side surface. The red and blue dots indicate the locations in k-space of the spin-polarized EDCs. (b) Intensity plot of the band structure along k_a and its MDC curvature plot. The red and blue lines indicate the k_a locations of the spin-polarized EDCs. Due to the quasi-1D feature of the Fermi surface, the band structure at $k_b \sim 0$ and $k_b \sim 10$ in (a) are basically the same. (c - e) Raw spin polarization curves along b, a and c directions measured at the indicated locations in (a) and from different samples. The polarization curves at the same k_a are almost the same (independent of k_b locations or samples). So the curves are just distinguished by their k_a values with red and blue colors. (f - h) Spin polarization curves along b, a and c directions, obtained by averaging the same-color curves in (c - e), respectively. Since the background part (< -0.15 eV) should show no spin polarization, we subtracted a constant for all the curves to remove the background spin polarization.

Here we discuss the details of the analysis on the spin-resolved data in Fig. 2f - 2i. We measured two pairs of EDCs (Supplementary Fig. 4a) by spin-resolved ARPES from three different samples. The k_a locations are also shown in Supplementary Fig. 4b. The spin polarization curves along the b, a and c directions are displayed in Supplementary Fig. 4c - 4e. In each panel of Supplementary Fig. 4c - 4e, the five curves with the same color (blue or red) are taken at two different k_b ($k_b \sim 0$ and 10, indicated in Supplementary Fig. 4a) from three samples (one sample at only $k_b \sim 0$, in total 6 - 1 = 5 curves). These five curves show no obvious difference, confirming the repeatability of the measurements. We notice that the spin polarization curves in the background range (< -0.15 eV) are not zero but have offsets. Such constant polarization in the background may come from the asymmetry of the spin + and spin - channels, or may come from the matrix element effect in the photoemission process, which is often observed in spin-resolved ARPES [3, 4]. We averaged the five polarization curves with the same color in Supplementary Fig. 4c - 4e, and subtracted the resulting curves by a constant to remove the background polarization. The results are displayed in Supplementary Fig. 4f - 4h. The spin is polarized along b direction, consistent with the calculations in Fig. 2j.

Supplementary Note 5: Measurements of tensile strain

The tensile strain of the sample was controlled by applying tensile strain to the substrate on which the sample was mounted. We separately measured the actual values of tensile stain applied to the substrate and the sample, and found consistency between the two. Most importantly, the strain values directly determined agree with that estimated from the band gap measured by ARPES.

We measured the tensile strain applied to the BeCu substrate with strain gauges (KFGS series from Kyowa Electronic Instruments, which can only be used for tensile strain). The strain was induced by increasing the torque on the screw in our device, which was monitored by a digital torque screwdriver. The obtained strain-torque curve is plotted in Supplementary Fig. 5a; we did two tests, and confirmed the two curves to be similar. The maximum torque we can apply is limited by the strength of Torr Seal used to fix the substrate to our strain device; in our setup, Torr Seal generally breaks at an applied torque between 0.2 and 0.3 N·m. This value may be slightly different, depending on the condition of Torr Seal, which is daubed onto the device by hand. In test 1, Torr Seal broke when torque > 0.195 N·m, whereas it broke when torque > 0.25 N·m in test 2. In the experiment shown in Fig. 3, we applied ∼ 0.2 N·m torque to the screw, which corresponds to a tensile strain of about 0.36%, according to the strain curve in Supplementary Fig. 5a. This value is basically consistent with the value 0.3% estimated from the band gap in ARPES (see the main text).

We also prepared one sample on the tensile strain device, applied 0.21 N·m torque to the screw, and measured the X-ray diffraction (XRD) peaks of the sample. The (080) and (170) peaks are obtained for the sample without and with strain, and these are displayed in Supplementary Fig. 5b. The peak positions and the corresponding lattice parameters are listed in Supplementary Table I. We found the strain of 0.42% is applied along the chain (a direction) on the sample; very importantly, this result agrees not only with the strain of the substrate measured by strain-gauge, but also with that estimated from the band gap in ARPES, all of which give ∼ 0.3 - 0.4 %, when applying the torque of ∼ 0.2 N·m to the screw in our strain device.

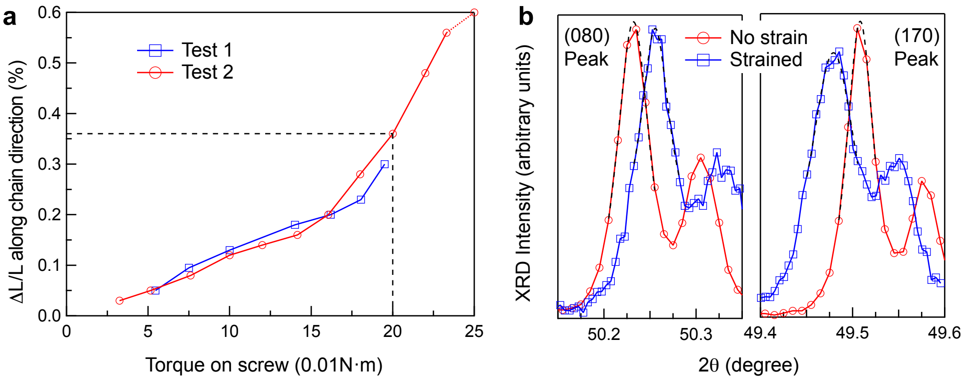

Supplementary Figure 5. **Strain measurement.** (a) Tensile strain on the substrate measured with a strain gauge. (b) XRD measurement on a sample with tensile strain (0.21 N·m torque on the screw).

Supplementary Table I. Parameters from XRD measurement

	2θ (080)	2θ (170)	b	a
No Strain	50.2319°	49.5083°	14.5128 Å	3.9817 Å
Strained	50.2556°	49.4792°	14.5064 Å	3.9986 Å

Supplementary Note 6: Restoration of the band structure after releasing the strain

The thin flakes we measured are too thin to cleave twice. Thus, to check if the band structure is reversible after the strain was released, we need a strain device tunable *in-situ*. Due to the limitation of our ARPES setup (location of a screw driver and the torque we can apply with it), the device shown in Fig. 3a was not suitable for such purpose. Hence, we used another strain device adjustable *in-situ* to check whether or not the tensile strain effect on the band structure of $ZrTe_5$ is reversible (Details of the strain device can be found in Ref. [5]).

In Supplementary Fig. 6a and 6c, we show the band structure obtained by strain cycle measurements for two samples: left to right panels each plot the data with no strain, $\sim$ 0.5% tensile strain, and strain released, following the experiment sequence. With $\sim$ 0.5% tensile strain (middle panels in Supplementary Fig. 6a and 6c), the band gap is enlarged (62 - 66 meV) from the original values ($\sim$ 30 meV), and then the band gap restores to its original value when the strain is released (right panels). This reversible behavior is clearly demonstrated in Supplementary Fig. 6b and 6d by plotting the EDC peaks on the horizontal axis which is the relative energy to the top of the valence band; the peak shift with strain and the restoring of it to the original position is clearly shown. Therefore, we conclude that the strain effect on the band gap is reversible. In passing, the spectra got weaker in intensity and broader than the original ones after releasing strain because of the surface aging of the samples: we transferred the samples to a chamber with a worse vacuum to release the strain and the vacuum became even worse when adjusting the screws to release the strain.

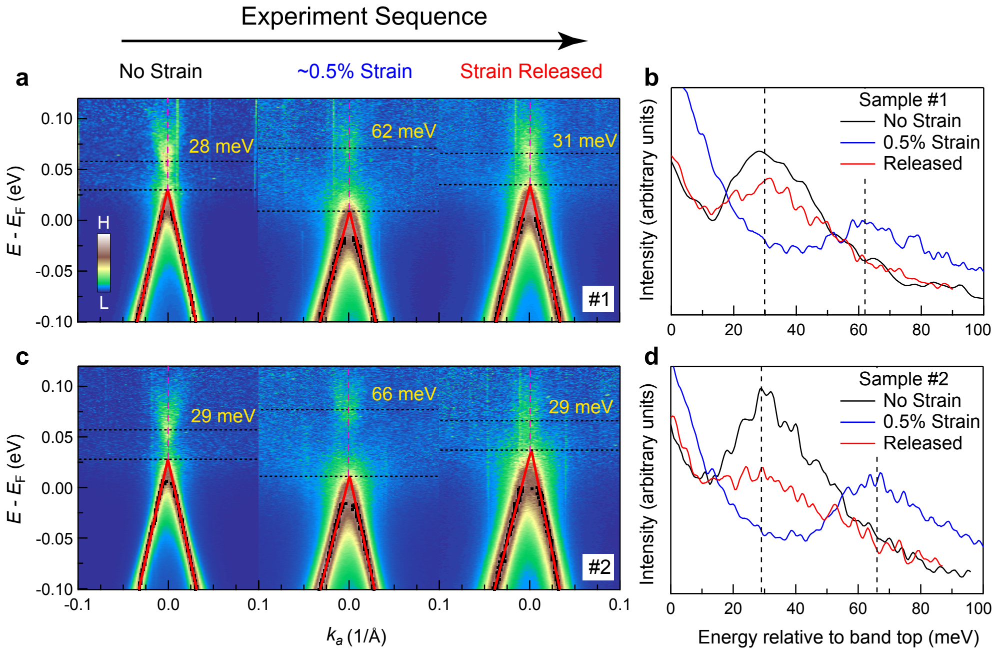

Supplementary Figure 6. **Band structure change with a tensile strain cycle.** (a) Band structure taken with zero strain, 0.5% tensile strain and zero strain for sample #1. The black dots and the red solid lines are the same as the ones in Fig. 1h. (b) EDCs at the cuts indicated by pink dashed lines in (a). The top of the valence band is used as the reference of zero energy. (c,d) Same as (a,b), but for sample #2.

[1] Zhang, Y. *et al.* Electronic evidence of temperature-induced Lifshitz transition and topological nature in $ZrTe_5$. *Nat. Commun.* **8**, 2367 (2017).
[2] Xiong, H. *et al.* Three-dimensional nature of the band structure of $ZrTe_5$ measured by high-momentum-resolution photoemission spectroscopy. *Phys. Rev. B* **95**, 195119 (2017).
[3] Heinzmann, U. & Dil, J. H. Spin–orbit-induced photoelectron spin polarization in angle-resolved photoemission from both atomic and condensed matter targets. *J. Phys.: Condens. Matter* **24**, 173001 (2012).
[4] Okuda, T. *et al.* Experimental Evidence of Hidden Topological Surface States in. *Phys. Rev. Lett.* **111**, 206803 (2013).
[5] Lin, C. *et al.* Visualization of the strain-induced topological phase transition in a quasi-one-dimensional superconductor TaSe3 (2020). arXiv:2009.06353.
